# Thorny entanglements: feminism, eugenics and the Abortion Law Reform Association’s (ALRA) campaign for safe, accessible abortion in Britain, 1936–1967

**DOI:** 10.1017/mdh.2024.4

**Published:** 2024-01

**Authors:** Susanne Maria Klausen

**Affiliations:** The Pennsylvania State University, Department of Women’s, Gender and Sexuality Studies, University Park, Pennsylvania, 16802-1503, US and the University of Johannesburg, Department of History

**Keywords:** Abortion, ALRA, Eugenics, Disability, Feminism, Transnationalism

## Abstract

For the past two decades anti-abortionists in the Global North have been aggressively instrumentalising disability in order to undermine women’s social autonomy, asserting, falsely, there is an insuperable conflict between disability rights and reproductive rights. The utilisation of disability in struggles over abortion access is not new, it has a history dating back to the interwar era. Indeed, decades before anti-abortionists’ campaign, feminists invoked disability to expand access to safe abortion. This paper examines the feminist eugenics in the first organisation dedicated to liberalising restrictive abortion laws, the Abortion Law Reform Association (ALRA), established in England in 1936. ALRA played a vital role in the passage of the Abortion Act 1967 (or the Act) that greatly expanded the grounds for legal abortion, a hugely important gain for women in Britain and beyond seeking legal, safe abortions. In addition, the Act permitted eugenic abortion, which also had transnational effects: within a decade, jurisdictions in numerous Commonwealth countries passed abortion laws that incorporated the Act’s eugenics clause, sometimes verbatim. This essay analyses ALRA’s role in codifying eugenics in the Abortion Act 1967 and argues that from the outset, ALRA was simultaneously a feminist and eugenist association. Initially, ALRA prioritized their feminist commitment to ‘voluntary motherhood’ in their campaign whereas starting in the 1940s, they subordinated feminism to negative eugenics, a shift that was simultaneously strategic and a reflection of genuine concern to prevent the birth of children with disabilities.

In the vast historiography on the global eugenics movement there is far more research on events up to 1945 than on the postwar decades. To a great extent, this is because of a longstanding interpretation of eugenics as a coherent, self-styled scientific theory of heredity harnessed to past exclusionary projects, most prominently nationalism. Eugenics was perceived as a program of selective breeding aimed at preventing individuals with undesirable traits, the ‘unfit’, from having offspring (negative eugenics) and encouraging those with desirable traits to have children (positive eugenics) in order to improve the quality of the nation or race, a project based on the belief that social traits like moral tendency and mental ability are biologically based and heritable. Then, according to this view, eugenics abruptly ended when Nazi atrocities came to light: once the world learned that Nazi eugenics culminated in the systematic destruction of millions of Jews as well as of Roma, Sinti and other ‘racial enemies’ of the German nation, eugenics was fatally discredited. This narrative goes far in explaining why there is still ‘an overall disinclination among historians to research eugenics after 1945’.^1^

In the past two decades, this periodisation has been steadily undermined by historians and disability scholars who convincingly argue eugenics should be understood not as a discredited, delusional and defunct science but as an ongoing biopolitical regime directed by an ‘evaluative logic’.^2^ By centering *selection* – the evaluative logic that ranks and values some people over other people – discursive continuities in eugenics before and after 1945 become visible.^3^ Probably the clearest example is reprogenetics, what some researchers call the ‘new’ eugenics (itself a contested term), responsible for today’s prenatal diagnostic tests designed to detect unwanted genetic traits that cause mental or physical disability.^4^ Far from fading away after 1945, therefore, eugenics should be understood as having what Alison Bashford characterizes as an ‘openly continuous history’ of transformation in policy, science, technology and politics.^5^ In short, proponents of a ‘long’ history of eugenics emphasise continuity over discontinuity in eugenic thinking over a long twentieth century.

One important area of continuity that is beginning to receive serious scholarly attention is campaigns to liberalise abortion law. Historians are painting a disturbing picture of how, after World War II and especially in the 1960s and 1970s, abortion law reformers lobbied for expanded access to safe abortion on grounds of foetal impairment, in the process deploying negative stereotypes about disability elaborated by eugenics movements in the interwar period.^6^ For example, the first law on abortion passed after the war was the Japanese Eugenic Protection Law 1948, proposed by doctors, socialists and birth control activists, that permitted abortion to prevent the birth of ‘eugenically inferior offspring’.^7^ In the US context, Johanna Schoen has shown how, ‘In their fight for abortion reform, health and welfare officials across the country turned to the same financial and eugenic arguments that justified eugenic sterilisation policies’ enacted in the first half of the twentieth century, and Mary Ziegler delineates how organisations like the Planned Parenthood Federation of America and the National Association for the Repeal of Abortion Laws ‘used fears of foetal disability and defect as a key reason to reform abortion laws’.^8^ I have highlighted how demands to include foetal abnormality as a ground for legal abortion were prominent in New Zealanders’ fight for abortion law reform in the 1970s.^9^ And Dagmar Herzog demonstrates how campaigns for abortion rights in Europe in the 1960s and 1970s were ‘saturated by references to disability’, some disturbingly reminiscent of Nazi discourse of just a few decades earlier.^10^ Evidently, she concludes, it was ‘quite hard to unlearn eugenic thinking’ for decades after the Holocaust.^11^

In all these cases, historians make clear reformers were fighting for expanded abortion access at a time when there was little to no public or political support for women’s right to control their reproductive sexuality whereas patronising and cruel assertions about disabled people as tragic objects of pity and social burdens were widespread, immediately comprehensible and useful.^12^ The effectiveness of discriminatory discourses about disability was thanks in large part to the labour of interwar eugenics movements that were intensely hostile to disabled people, considering them biologically tainted and ‘dysgenic’, meaning harmful to future generations.^13^ These studies are illuminating the thorny entanglements between eugenics, feminism and campaigns for abortion law reform, but much remains to be discovered. Indeed, Bashford calls this ‘[a]rguably the most overlooked trajectory’ of the long history of eugenics.^14^

This essay contributes to the emerging historiography by focusing on the British fight for abortion law reform that began in 1936 when a group of feminists established the Abortion Law Reform Association (ALRA) and, after decades of persistent campaigning, culminated in the passage of Abortion Act 1967. Abortion Act 1967 (or the Act) created exceptions to the criminal offences for abortion contained in sections 58 and 59 of the Offences Against the Person Act 1861 and section 1(1) of the Infant Life Preservation Act 1929 (sections which persist).^15^ It contains two clauses specifying when doctors could lawfully perform abortion. Section 1(a) permits abortion when a woman’s pregnancy risked her life or her physical or mental health or that of any of her existing children. This clause, what I call the woman-centred section, greatly expanded access to state-funded medical abortion and made procuring abortion far easier, safer and less punitive, reasons for which Abortion Act 1967 has been rightly hailed as a major victory for British women.

Perhaps less widely known, section 1(b) allows abortion on grounds of foetal impairment, stating abortion is permitted when ‘there is a substantial risk that if the child were born it would suffer from such physical or mental abnormalities as to be seriously handicapped’.^16^ This section, which is still in the Act, was commonly called the ‘eugenics clause’ by both contemporary and subsequent commentators, including feminist researchers, health advocates, politicians and anti-abortionists.^17^ The ubiquity of the term in the 1960s and 1970s is not at all surprising in light of Herzog’s point that eugenic discourse about disability was still widespread in Europe for decades after the Holocaust. In Britain, too, the backlash against eugenics ‘was considerably delayed’ after the war.^18^

There is little research on how, precisely, eugenic abortion came to be codified in the Abortion Act 1967 which is surprising given how often it has been challenged over the years by anti-abortion organisations and individuals arguing it is discriminatory against disabled people.^19^ What has been made clear, not least by ALRA members themselves, is the role of the thalidomide crisis in shifting public and political opinion about abortion.^20^ In the early 1960s, the birth of thousands of babies with severe physical deformities as a result of the untested drug thalidomide was shocking and provoked a rapid shift in public perception of abortion from a disreputable topic tainted by sexual promiscuity and criminality to a public health necessity.^21^ After thalidomide, there was an upsurge in demand for legalising abortion when wanted to prevent the birth of a disabled child. What has been almost completely overlooked, however, is ALRA’s promotion of eugenic abortion years *before* thalidomide: starting in the early 1950s, ALRA worked in alliance with the still-active Eugenics Society to legalise abortion on grounds of foetal impairment.^22^

What has certainly been overlooked is the eugenic clause’s important transnational impact. Lawmakers in numerous Commonwealth jurisdictions incorporated it, often verbatim, in abortion legislation passed after 1967 – in most cases without including the woman-centred clause. Between 1967 and 1977, six jurisdictions in the Commonwealth included it in newly crafted abortion laws.^23^ In addition, South Africa (which withdrew from the Commonwealth in 1961) adopted an extended version in its first statutory law on abortion, the misogynistic Abortion and Sterilisation Act 1975.^24^ And in 1978, the New Zealand government amended its abortion law specifically to include it, almost verbatim.^25^ The rapid uptake suggests that the Abortion Act 1967 crystallised, named and legitimised a preexisting and pervasive, if previously undefined, attitude of intolerance towards disability in many countries. Thereafter, other jurisdictions around the world named foetal impairment as an indication for legal abortion: according to the United Nations (UN), by 2011, about 60 percent of the world’s countries permitted abortion on grounds of foetal impairment.^26^ That foetal impairment has so frequently qualified as one of a tiny number of indications for legal abortion is a clear example of the continuing power of eugenic thinking about disability after 1945.

Why and how did ALRA, the world’s first pressure group dedicated to reforming abortion law, established by feminists wanting to expand access to safe abortion for women, transform into a committed advocate of eugenic abortion in postwar Britain? This essay sets out to answer this question and is organised in three parts. The first section reviews the historiography on ALRA and offers an explanation for the lack of sustained analysis of the role of eugenics in their campaign from 1936 to 1967. Part two examines the formation of ALRA and the initial, exhilarating feminist phase of their campaign until the outbreak of war. Part three traces the emergence and deployment of eugenics in the campaign after 1945 and until the thalidomide crisis that forever changed perceptions of abortion in Britain. The essay is based on a close examination of the campaign’s discourse as well as the writings and actions of the three founding members who remained ALRA leaders until their deaths: Stella Browne (1880–1955), Janet Chance (1886–1953) and Alice Jenkins (1886–1967). Ultimately, it demonstrates ALRA was always a feminist eugenic pressure group; however, there was a dramatic shift over time in their campaign’s emphasis from maternal feminism to eugenics. As in other national contexts, ALRA found the postwar era hostile to feminism and so they leaned on eugenic thinking instead. This was not a purely tactical, pragmatic move, however: while always maintaining their feminism, by the 1950s ALRA firmly believed in the morality of abortion on grounds of foetal impairment. The conclusion offers a brief reflection on the legacy of feminists’ past embrace of eugenics for the ongoing struggle to expand access to safe abortion in Britain and beyond.

## Locating eugenics in the historiography on ALRA

To date, researchers generally take one of two approaches to assessing ALRA’s ideology. On the one hand are studies that emphasise their maternal feminism, meaning their commitment to the principle of ‘voluntary motherhood’, and downplay or disregard their eugenic thinking.^27^ On the other hand are studies that conclude ALRA was solely dedicated to a conservative, even ultraconservative, social agenda. Among the latter, one argues that initially, ALRA’s ‘primary concern was maintenance of the family’ and that their focus shifted over time to eugenics; the word ‘feminist’ is never used.^28^ Another study outright rejects ALRA’s feminist proclamations of deep concern for working-class women’s health and wellbeing as disingenuous. This is the conclusion of Ann Farmer, the only historian thus far to have conducted an in-depth study of the whole of the British abortion campaign. In her book *By Their Fruits: Eugenics, Population Control, and the Abortion Campaign* (2008), Farmer reduces ALRA to a mere branch of the Eugenics Society, whose classist, racist and ableist efforts over the course of the twentieth century to prevent the proliferation of the ‘unfit’ she discusses at length.^29^ Farmer concludes ALRA was a harmful eugenic organisation that was the ‘fruit of movements dedicated to the eradication of the disabled and the control of the poor and non-white’ and that ‘[t]hroughout its many evolutions, the English abortion campaign, true to its philosophical origins, has placed eugenic concerns above all others’.^30^

Based on my own reading of ALRA’s records and its leaders’ public pronouncements and published texts, I agree with Farmer’s assessment that ALRA subscribed to eugenics and her contention that some ‘[a]bortion reform histories have emphasised the feminist and radical character of campaigners, [while] mostly overlooking their eugenics and Malthusian connection’.^31^ But her claim that ALRA’s feminism was a ruse, essentially ideological camouflage for their ‘real’ aim of legalising eugenic abortion, is wholly incorrect. For example, Farmer states, wrongly, ‘while arguing for abortion on the basis of backstreet abortion…[ALRA in the 1930s was] overwhelmingly interested in eugenics and population control…The campaigners’ emphasis on maternal health, maternal mortality, the desperation of pregnant women, and the welfare of children implies a compassionate motivation’, when in fact these were merely ‘tactical’ arguments.^32^ She even goes so far as to assert ‘the English abortion campaign actually originated in movements opposed to feminism, namely, eugenics and population control’.^33^ As shown below, this damning assessment is grossly inaccurate.

There are at least three reasons for the paucity of studies on the origins and effects of the Abortion Act 1967’s eugenics clause. The first relates to the periodisation of eugenics that has resulted in the ‘general disinclination to study eugenics’ after 1945, discussed above.

The second reason is the overwhelming tendency to examine the Act within a national framework. As already demonstrated, the eugenics clause was taken up, sometimes verbatim, by numerous other jurisdictions across the Commonwealth, developments rendered invisible when strictly limiting analysis of the campaign to the British context. Utilising a transnational lens reveals more than just the post-1967 world travels of the eugenics clause, it also reveals how ALRA was from the start influenced by both international developments in abortion law and the flourishing transnational eugenics movement.^34^ From the 1930s onward, ALRA followed abortion politics in other countries, sometimes with the specific goal of learning which ones included a eugenics clause and how such clauses were worded.^35^ This was a task assigned as early as 1936 to one of ALRA’s co-founders, Stella Browne, who tracked events elsewhere for members.^36^ Browne was already doing this anyway: in 1935, for example, she wrote approvingly of proposed abortion legislation in Czechoslovakia that advocated abortion when ‘it is likely that the child will be gravely tainted physically or mentally’, commenting this was ‘[a]n excellent principle, but somewhat difficult in practice’.^37^ (Aside from the eugenics question, a transnational analysis also reveals how very important, and still underestimated, an impact the Abortion Act 1967 and ALRA had on women’s struggles to access safe abortion in countless countries. The Act was a lifeline for women who were unable to obtain safe abortions in their own countries and had the resources to travel: after the law’s passage, thousands began travelling to Britain in search of medical abortions,^38^ an unexpected development that alarmed ALRA who feared for the new law’s survival.^39^ Moreover, the Act was an inspiration to individuals and organisations campaigning for abortion law reform in dozens of countries, with many reaching out to ALRA with requests for advice, contacts, research and campaign material. Sharply aware of the symbolic importance of the Act, ALRA did their best to help.^40^)

Finally, it is another example of the marginalisation of disability in the historiography of eugenics, including British eugenics. Historians have long discussed how the British eugenics movement was unusual if not unique for being a classist phenomenon in contrast to its German, American and other counterparts that were obsessed with race.^41^ As Donald Mackenzie writes, British eugenics ‘is not to be understood in terms of preoccupation with Jews, Blacks or immigrants’ although undoubtedly British eugenists like Britons in general held racist views. Instead, it should be viewed primarily as a movement that ‘served both to legitimate the social position of the professional middle class and as an argument for its improvement’.^42^

However, disability scholars argue that regardless of national context, the centrality of prejudice and hostility to disabled people has been vastly underestimated in historical research on eugenics. David Mitchell and Sharon Snyder attribute the marginality to ongoing ‘cultural ambivalence about the status of disabled people’ and ‘a continuing social reluctance to imagine disability as a valued aspect of the human biological continuum’.^43^ A proper grasp of the meaning of eugenics, therefore, requires understanding it as a politics of *normalisation.* ^44^ Michael Rembis asks, ‘What if one began an assessment of eugenics with the assumption that it was infused not with a more or less virulent racism, but with a more or less virulent ableism’ that was always gendered, classed and racialised? ‘What if, in other words, the history of eugenics was defined not by the genocidal actions of Nazi Germany but by its more common manifestations in other parts of the world?’ By common manifestations Rembis is referring to the long twentieth-century practices of ‘segregating, sterilising, and generally restricting the world’s disabled population’ in order to ‘relieve suffering, reduce welfare costs, and eliminate poverty, immorality, and crime …’. Despite national differences, he concludes, eugenists ‘remained generally united in their desire to rid the globe of burdensome “defectives.”’^45^

In other words, to eugenists everywhere and over time, disabled people were the prime example of the ‘unfit’ to be pitied, feared and devalued, nowhere more so than in Nazi Germany where approximately 400 000 mentally and physically disabled Germans were coercively sterilised and at least 230 000 were considered ‘life unworthy of life’ and systematically murdered.

## Advocating ‘voluntary motherhood’: 1936 to World War II

From 1936 until World War II, ALRA advocated abortion access in the name of ‘voluntary motherhood’, a radical endeavour in a society such as Britain’s whose laws and norms staunchly opposed women having control over their reproductive sexuality. Some of ALRA’s *leaders* endorsed eugenics elsewhere, although what they meant by it is far from clear, but the group’s *campaign* never deployed eugenic discourse.

ALRA was established in February 1936 by seven middle-class women who strenuously opposed legal obstacles to safe abortion. The catalyst was knowledge that poor and working-class women lacked access to private abortions performed by trained medical doctors, a service that middle- and upper-class women could buy. Based on their experiences as medical doctors, birth-control activists and sex-reform radicals, the founders knew that women who lacked the requisite funds for medical abortions were forced to either bear unwanted children or else have unsafe clandestine abortions and that the latter was contributing to the high rate of maternal mortality alarming authorities at the time.^46^ From its inception, the official goal of ALRA was to ‘repeal the present law’ and substitute it with one ‘freeing the medical profession from all legal restrictions, except those required by medical or humanitarian restrictions’.^47^ ALRA’s constitution proclaimed ‘that the widespread practice of secret abortion, whereby unqualified persons, endanger the lives of pregnant women, will not be ended by the present abortion laws’ and advocated that ‘abortion by qualified practitioners be legalised within such limitations as may be considered advisable’. Specifically, they wanted the repeal of Section 58 of the *Offences Against the Person Act* (1861) that criminalised intentional miscarriage and the *Infant Life Preservation Act* (1929) that carried the potential of a life sentence for someone who kills a ‘child’ who is ‘capable of being born alive’.^48^

ALRA was of particular importance to Stella Browne, Janet Chance and Alice Jenkins. All were feminists who had been involved in the Labour Party and the campaign for accessible birth control in 1920s and 1930s, experiences that educated them in the harsh reality of being a poor woman desperate to avoid pregnancy. Chance had been on the executive committee of the Society for the Provision of Birth Control Clinics and a volunteer at the Walworth Birth Control Center and the Directory of Sex Education Centre. Jenkins had been a secretary of the Ealing Branch of the National Council of Women, a chair of the Ealing Branch of the National Birth Control Association and a volunteer at the Goswell Women’s Welfare Clinic.^49^ And Browne was a tireless sex-reform activist and advocate for women’s sexual liberation with ‘an exacting schedule of lectures to women’s, working-class, and secularist organisations’^50^ and author of numerous provocative texts demanding ‘social and sexual freedom for women’.^51^ Prior to 1936, Chance, Jenkins and Browne had all been outspoken critics of England’s stifling and misogynist attitudes to sex and morality.^52^ As Chance wrote in 1931, conventional attitudes to sex caused women’s ‘personal and bodily slavery’ and added their emancipation would be incomplete without easy access to contraception and safe abortion.^53^ All three women wanted a new morality for Britain, one free from what Browne called the Christian ‘superstition’ and its ‘doctrine of the uncleanness of sex’.^54^

The three women helped blaze a feminist path for public criticism of existing laws and attitudes to abortion. As early as 1915, Stella Browne publicly called for abortion on demand, the first Briton to do so. Alice Jenkins delivered her first public address on the topic of abortion – what she called ‘that terrible word’ – in a speech in the early 1930s, a talk that emphasised the dangerousness of pregnancy for many women. She opened her speech describing the agonising experiences of three women she learned about while volunteering for a woman’s organisation. One woman was poor, one was wealthy and one was from the middle classes; the first woman died in childbirth, the second died from self-induced abortion and the third suffered an excruciatingly painful, unwanted pregnancy but survived. Each had practiced a form of birth control that had failed. Jenkins then said the following:

… being completely ignorant of the fact that termination of pregnancy could be procured cleanly and safely, I reached the incorrect conclusion that safer motherhood could be obtained by better facilities for the teaching of scientific conception control; and knowing that inquiries from patients were often met by evasive and even facetious replies by doctors, I helped my organisation to be the means of inaugurating a local B.C. [Birth Control] clinic – in the teeth of a small but powerfully organised sectarian opposition. This victorious struggle, however, brought about a curious result – the certainty that contraception was not a complete defence against unwanted pregnancy … Contraceptives were neither reliable nor cheap [and] [p]rivacy to adjust the appliances was almost impossible in overcrowded living conditions.

At about the same time, she continues, she

… heard that an acquaintance, the wife of a physician and mother of three young children, had been quietly and comfortably aborted of her fourth pregnancy by a competent surgeon. Having by this time learnt many horrifying details about unskilled abortion, this skilled termination seemed a revelation – an almost incredible way of escape from [the] welter of maternal suffering ….

Convinced that ‘refusal of motherhood’ was a ‘root cause’ of maternal mortality, Jenkins began writing letters to various publications on the issue, which is how she met Browne: as a result of writing letters, ‘other interested people – amongst them Miss Browne, our most courageous advocate – got into touch with me’, and soon thereafter they raised the subject of abortion at the 1934 Maternal Mortality Conference.^55^

At the start, ALRA’s campaign was infused with maternal feminist discourse, evident in their vigorous rejection of ‘forced motherhood’ and calls for its opposite, ‘voluntary motherhood’. The stance was both a common touchstone for members embodying different political perspectives and a strategic choice given the need to win public and political support for their controversial mission. As Stephen Brooke explains, deployment of maternal feminist rhetoric reflected the group’s concerns ‘about gaining legitimacy and respectability’.^56^ They were extremely active in the 1930s. Fuelled by excitement over authorities’ relative openness to abortion law reform, they lobbied Members of Parliament (MPs), wrote letters to newspapers, organised dozens of public meetings and more. In 1937, their membership was 200 and they had affiliated with twenty branches of the Women’s Co-operative Guild and other associations.^57^ They organised conferences and spoke to many organisations. Their first conference was held in May 1936, and between 1 May and 30 September 1937, members spoke at fifty-seven meetings and had another fifty-two planned for October to December.^58^

In 1937, ALRA submitted a memorandum to the Inter-Departmental Committee on Abortion, commonly called the Birkett Committee, appointed in response to alarming reports that illegal, unsafe abortion was a major factor in the high maternal mortality rate. In it they listed eight reasons for liberalising the law, all of which reflected a maternal feminist focus on women’s health and the welfare of the family: ‘the maintenance of an adequate standard of life for the family as a whole’; pregnancy resulting from incest, rape or criminal assault; immature age; unmarried status; loss of employment; ‘the fear of handing on some trait which has proved disastrous in the family history of husband or wife’; loss of a (male) wage-earner; and risk of failure of a woman’s ‘strength and happiness’.^59^ Browne went considerably further when making her solo presentation to the Birkett Committee (as an individual rather than as a representative of ALRA), declaring availability of abortion on demand a precondition for women’s liberation. She stated, ‘I can only speak for myself, but … I aim at making life more bearable and more interesting and better and bigger for the majority of women’, and she freely admitted to once having procured an abortion herself.^60^ Until the end of her life, Browne never wavered from her famous, unequivocally radical feminist belief that abortion ‘should be available to any woman without insolent inquiries nor tangles of red tape, for our bodies are our own’.^61^

After the presentation to the Birkett Committee, Janet Chance wrote to ALRA’s members saying they need to choose a strategy to pursue going forward. It was already established, she stated, that ALRA believed in the principle of ‘voluntary parenthood’ and desired to find a solution to the ‘maternal health’ crisis, but to do so they needed to pick one of two approaches, the first mainstream and the second radical. ALRA could ‘set out to lead all progressive thought on A.L.R. [Abortion Law Reform] and become tactical, political and diplomatic in advocating a compromise here or a partial reform there, in order to get some immediate reform as rapidly as possible’. Or they could ‘set out to advocate certain fundamental principles … which we believe to be fundamental to health and happiness and that we do so even at the cost of losing the support inside A.L.R.A. of the half-way reformer and of those more acceptable to conventional opinion’. Chance advocated the latter, stating:

I hold that if in the early stages of the Birth Control movement we had allowed short-range and diplomatic considerations to guide us, the doctors would not today be as free as they are to give us their invaluable services in birth control work. The community had to free them there. So it still has to free them in abortion work.

She proclaimed theirs ‘is not a medical or legal fight; it is an ethical one … Nothing but the re-orientation of public opinion and the making vocal of the opinion we find inarticulate in the lives of women will settle it. That is the task I am prepared to work for’.^62^

Chance also offered members advice on how to speak publicly about abortion law reform, recommending they emphasise the following points: ‘the seriousness of secret abortion’; that there was ‘One law for the rich and one for the poor’; that ‘woman’s opinion is at present unheard and considered unimportant’; and, regarding moral objections, ‘abortion will make marriage more tolerable and therefore stabilise it’. When asked what ALRA wanted, speakers should say, ‘The legalisation of abortion for wide medical reasons …: for economic reasons: for social and personal reasons’.^63^ Members subsequently engaged in a whirlwind of public advocacy.^64^

In these heady years, the campaign never advocated eugenics. Their primary, passionate concern was the welfare of women and their families, as is clear in an exchange between Norman Birkett, chair of the Inter-Departmental Committee on Abortion, and Dr. Joan Malleson, an ALRA co-founder. When Birkett asked Malleson, ‘Have you considered how frequently a fourteenth or fifteenth child in a family has become a great man?’ she shot back, ‘Have you considered how rapidly the maternal death rate rises after the fifth child?’^65^

The absence of a discourse of eugenics is despite the fact that at least Browne, Chance and Jenkins subscribed to the new science-based social movement. They were all members of the Eugenics Society (or the Society): Jenkins was elected a Fellow in 1933, Chance joined in 1939, and both remained members until their deaths. Browne joined in 1938 but her membership lapsed in 1942, probably because of chronic financial hardship.^66^ In addition, all three were members of the World League of Sexual Reform, whose manifesto called for the ‘Application of the knowledge of Eugenics towards the improvement of the race through Birth Selection (Encouragement of propagation of the fit and gifted, and sterilisation for the unfit)’, and of the antireligion, proscience Federation of Progressive Societies and Individuals that advocated the legalisation of abortion and sterilisation of ‘the congenitally unfit’.^67^ In addition, Browne was an active member of The Malthusian League that promoted birth control to reduce poverty caused by ‘overpopulation’ and was aligned with the eugenic goal of preventing ‘unfit’ parents from having children.

Their membership in eugenic organisations is hardly surprising given British feminists’ widespread attraction to eugenics in the interwar era. Except in countries where eugenics was promoted by the far right, such as Germany, feminists often had a positive, unsuspicious attitude to the new self-styled scientific movement. Greta Jones explains the overlap in movements: ‘Eugenics was about manipulating women’s reproductive power’ and it was the politicisation of reproduction that attracted feminists.^68^ As Ann Taylor Allen observes, eugenics had a ‘formative impact on some of the most important feminist campaigns of the twentieth century – including those for maternal and child health, birth control, and family allowances’.^69^

But mere membership in these organisations does not automatically explain what they thought eugenics meant. Historians have amply demonstrated it was never a fixed idea or program because its meaning was always contextual, contested and unstable, and social movements promoting eugenics attracted adherents from across the political spectrum.^70^ As Lesley A. Hall asserts, ‘Eugenics is too often assumed to have been a monolithic and clearly understood ideology, stable over time and predictive of particular attitudes and sympathies in its adherents, whereas there was no one eugenics either in beliefs or policy implications’.^71^

Certainly, as educated women of the middle classes they shared elites’ understanding of eugenics as an applied form of science, which to them specifically meant a reason-based force they hoped would break down the harmful conventional morality and social problems plaguing their society. This is in keeping with Taylor Allen’s assessment that British feminists generally favoured eugenics because it dovetailed with their ‘cheerful confidence, derived from their background in the Fabian Socialist movement, in the efficacy of rational planning in all areas of life and politics’.^72^ Like eugenists everywhere, they also found that eugenics made sense as a ‘biological way of thinking about social problems and social change’.^73^ They seem to have shared in the widespread belief that ‘feeblemindedness’, a term ‘associated with people with physical, mental and sensory disabilities’, was a biologically based condition that was dragging down British society. This was a powerful prejudice in British society at the time, one that fuelled the systematic discrimination against people believed afflicted.^74^ By extension, they likely considered disabled children undesirable because their care fell disproportionately to mothers. Finally, it appears in their repeated assertions that eugenics would improve the ‘race’, a multivalent term in the interwar era, they all took for granted that the British race was, and likely should remain, white. In stark contrast to feminists such as their contemporary Sylvia Pankhurst, they never evinced awareness or concern regarding Britain’s racist underbelly or its imperial ‘mission’.^75^

At the same time, the women interpreted the main goal of eugenics differently. For Browne, it was predominantly a set of ideas about population health to mobilise to feminist ends, meaning as justification for women’s sexual and reproductive freedom. She repeatedly asserted the ‘race’ would benefit from children being conceived by women fulfilling their sexual desire. And she passionately believed every child should be wanted; children born to women who did not want them, she claimed, were emotionally damaged as well as physically inferior. In numerous passages she appears to draw on both the old folk belief that children begotten in love rather than tedious marital sex are superior and the new idea that ‘race degeneration’ was caused by traditional morality.^76^ (The famous British birth-control activist Marie Stopes also argued that unwanted pregnancy had an adverse effect on women’s hormones, affecting children’s health.^77^) In 1912, Browne wrote about ‘the neurotic child’ produced in a marriage in which women hated the sex. Such children are ‘cursed with self-consciousness and self-distrust, hypersensitive, unbalanced, often afflicted either with morbid egotism or complete atrophy of the will’.^78^ Five years later, she wrote ‘the magic forces that will revitalise and transfigure the race’ are ‘[a]bsolute freedom of choice on the Woman’s part, and intense desire both for her mate and her child’. She concludes her essay thus: ‘The eugenic aspect of love and sex has been very neatly summed up by a woman-poet, Anna Wickham: The world whips frank, gay love with rods; But frankly, gaily, shall ye get the gods’.^79^

Indeed, Browne joined the Eugenics Society despite having been publicly critical of the organisation’s obsession with class-differential fertility. In scathing letters published in the *Freewoman* in 1912, she expressed outrage at the Society’s members who ‘met … to decide who is to be born and who is not’.^80^ During World War I, she wrote:

If the Eugenics Education Society deserved its name, it would undertake in this country, the work that Margaret Sanger – to whom be honour and gratitude for ever – is doing in America. In view of the gross neglect of women’s interests as mothers and as citizens and of the lean years before us all, the demand for a higher birthrate is both impudent and inhuman. The underhand opposition to the spread of contraceptive information must be over come. The ineffably foolish laws penalising abortion must be abolished; they are one of the foulest remnants of the Canon Law.^81^

As Hall suggests, Browne likely joined the Eugenics Society to persuade the far wealthier organisation to support her cash-starved feminist causes for reproductive control. Similarly, she probably joined the Malthusian League because, at the time she joined, around 1912, ‘it was the only British body … explicitly committed to advocating the artificial limitation of births and providing information on the subject’.^82^

Jenkins’ and Chance’s ideas about eugenics in the 1930s are harder to determine, but they seemingly shared the Eugenics Society’s classist concern about differential birthrates. When establishing ALRA they consulted leading members of the Society, such as Lord Thomas Horder (president from 1935 to 1949) and C. P. Blacker (secretary from 1931 to 1952); cultivating the connection not only made sense ideologically, it also conferred respectability and accumulated influential allies. Another indication of Chance’s amenability to the Society’s elitist outlook was her friendship with Blacker, whom she called by his nickname ‘Pip’. Both women’s approval of the Society’s preoccupation with ‘population problems’ would explain why they so rapidly prioritised eugenics in ALRA’s campaign after 1945.^83^

## Embracing eugenics: World War II to 1967

After 1945, ALRA’s campaign became increasingly preoccupied with ‘population problems’, especially the birth of ‘defective’ babies, to the extent that by the mid-1950s ALRA was promoting eugenic abortion in highly discriminatory terms.

The outbreak of war abruptly halted the momentum of ALRA’s campaign. The war instantly eroded official and popular interest in maternal mortality and, by extension, abortion law reform. With Britain’s survival at stake, the political elite became preoccupied with a new ‘population problem’: underpopulation.^84^ Elites wanted more, not fewer, (white) Britons to maintain national strength.

ALRA was still a feminist organisation but responded to the conservative sociopolitical wartime context by subordinating their demand for voluntary motherhood to more socially acceptable arguments for abortion law reform. At a meeting in 1944, members began framing abortion in terms that would be beneficial to the ‘community’ rather than to women and their families. When discussing a draft bill on abortion to use for lobbying MPs, members considered using the following statement proposed, apparently, by Lord Horder who was both an ALRA member and president of the Eugenics Society: abortion should be lawful on ‘grounds of physical or mental health or at a time when it appears medically or socially desirable either in her own interest or that of the community that she shall not give birth to a child’. However, whilst acknowledging ‘the present state of population fears’, ALRA opted instead to send a compromise statement to newspapers: they agreed ‘the war has drawn attention to our need of a wise population policy’ and to ‘the importance of sound child-bearing’ *and* reaffirmed that ALRA ‘stands unreservedly for the principle of voluntary parenthood’. The letter continued in defensive terms, ‘if this is too much to ask of 1944, a reform of the abortion laws which will bring medical advice to the woman who considers her pregnancy in some special circumstances disastrous, is surely a measure of first rank importance which should be included in all population policies’.^85^

ALRA also sent a letter to the Royal Commission on Population, formed in 1944 to examine the causes and consequences of population trends, asking for an inquiry into the ‘harmful result on fertility of women through repeated unskilled abortions’.^86^ And, evincing a blend of the prewar defiant feminism and a new pronatalist discourse, Jenkins in 1943 and 1944 wrote letters to the *Eugenics Review* simultaneously excoriating misogynistic assertions that educating women stifles their desire to biologically reproduce and demanding the removal of ‘legal and economic disabilities of wifehood and motherhood’.^87^ But these actions did not revive official interest in abortion law reform. As Stella Browne reported in 1945, ‘It has not been easy to get a hearing for the case for abortion law reform … in wartime. My efforts to get any of the relevant questions put in Parliament … have failed’.^88^ ALRA’s membership also shrank drastically during the war.

As soon as the war ended, Jenkins and Chance tried to reactivate the campaign but they were now operating in a sociopolitical climate that was hostile to radical, progressive ideas. The 1950s was a bleak decade for feminism, one in which the nuclear family was valorised as the foundation of the new welfare state, and there was profoundly decreased discursive space for arguments for women’s reproductive control.^89^ In addition, racism was intensifying. White Britons developed imperialist anxiety about the weakening empire and population growth in former colonies, concerns mirrored domestically in the racist response to the immigration of hundreds of thousands of brown and black people from the colonies.^90^ As Britain lost its global economic superiority and entered a period of imperial decline, the need to rebuild national cohesion led to ‘questions of race [becoming] central to questions of national belonging’.^91^ As Clare Hanson observes, ‘Eugenic concerns with the health of the population overlapped with the aims of post-war reconstruction to a significant extent’.^92^ Indeed, according to Lucy Bland and Lesley Hall, ‘To some extent, ideas about the innate unfitness of certain elements of the community became even *more* acute once the postwar welfare state provided a safety net against the worst ravages of poverty …’. (my emphasis).^93^ This is evident in how ongoing assumptions about the innate ‘low quality’ of the poor informed government research and policies related to identifying and treating the ‘mentally deficient’ and ‘problem families’ in the 1950s and 1960s, a reactionary development that C. P. Blacker fostered with his report on the state of mental health services in Britain.^94^

Realising what they were up against, when ALRA met for the first time after the war Chance cautioned members as follows: ‘I advise a realistic attitude to the present state of public opinion, necessitating a slow approach to full legalisation, accompanied by education and gradual formation of a wise attitude to all that is involved’.^95^ This was a drastically different message from the one she delivered in 1937 and it signalled the end of the rebelliously feminist phase of their campaign.

The loss of Stella Browne’s active involvement was a crucial factor in ALRA’s postwar conservatism. Browne moved to Liverpool during the war and although she constantly mailed her ideas and opinions to ALRA, to the extent that at times she tested the patience of her London-based colleagues with her frequent missives, she inevitably fell out of touch with daily developments and discussion and was therefore unable to exert significant influence. Without her unbending countervailing radical feminism, it became easier for Chance, Jenkins and the handful of remaining members to adopt a mainstream, politically conservative discourse and strategy for eliminating legal barriers to abortion. In fact, ALRA consciously made the tactical decision to distance the campaign from Browne’s radicalism. As Jenkins later explained, they believed the ‘cause’, which continued as before to be about making safe abortion accessible to all women, would be better served by avoiding the ‘forthright’ feminist declarations of the sort Browne tended to make, such as it was a ‘woman’s right to abortion up to the viability of her child’.^96^

ALRA stalwarts Chance and Jenkins and the other remaining members of ALRA were demoralised but not defeated by the depressing new sociopolitical reality. In search of powerful allies, they turned to the two male-dominated groups still interested in abortion law reform: the medical profession and the Eugenics Society.^97^ Neither cared about women’s social and sexual liberation but both offered resources and support, albeit on the men’s terms.

After the creation of the National Health Service in 1948, which offered publicly funded medical care, and emboldened by the Bourne decision, doctors became increasingly amenable to performing abortions.^98^ Medical members of ALRA confirmed that hostility to abortion was waning. Dr. Eustace Chesser, for example, ‘emphatically’ told the ALRA executive in 1951 that the attitude of doctors was markedly changing.^99^ In 1952, Jenkins wrote to an ally: ‘There is good reason to believe that many doctors are now terminating pregnancy for serious health reasons without waiting until the patient is at death’s door’.^100^ From the early 1950s onward, therefore, ALRA embraced a medicalised discourse on abortion and focussed on fighting for a *doctor’s* right to perform abortions free from fear of prosecution rather than, as Browne would have put it, a *woman’s* right to have them.

After the war, the Eugenics Society became involved with devising methods to combat ‘world overpopulation’, and drawing nearer to the Society dovetailed with Chance and Jenkins’ increasingly classist, racist pronouncements about fertility issues at home and abroad.^101^ Already in 1946, Jenkins wrote a letter to the *Eugenics Review* arguing ‘this small island’ (Britain) would face chaos if the population grew much larger and stating ‘the root cause of the present Indian food shortage is over-breeding’, and calling for the creation of a Population Investigation Committee under the direction of the UN. She also expressed concern about differential fertility, stating that, while family allowance (introduced in 1946) was likely intended to ‘stimulate numbers’ during a period of anxiety about underpopulation, ‘Competent observers believe there are already signs of this result in the least desirable section of the community’.^102^ In 1949, Chance told ALRA members they ‘had some grounds for optimism’ because ‘it seemed to be at last dawning on the minds of people that there is a direct relationship between population and standards of living’, and approvingly shared a press cutting about Japan’s Eugenic Protection Law that reported, ‘following on recent reforms in the Japanese Abortion Law, their birth rate has been almost halved’.^103^

Thus, ALRA rapidly reoriented their campaign in alignment with that of the Eugenics Society’s. As Chance wrote the Society in 1950, she ‘always looked at the Birth Control Movement, the Eugenics Society and the Abortion Law Reform Association as three aspects of planned parenthood …’.^104^ This view reflected Blacker’s own sense of complementarity of the projects, all of which emphasised planning. As Blacker wrote in 1961, the objective of eugenics is

… identical with the objective of the Family Planning Association. According to this objective, the particle *eu* in eugenics is reflected not in single attributes of parents such as intelligence, health, physique, etc, but in a performance test…begetting and rearing a happy and well-adjusted family, the children being wanted and conceived by design … eugenicists favour the planned as against the unplanned family.^105^

In 1951, Chance and Jenkins called for a new abortion law devised along the lines of Sweden’s, passed in 1938, that had a eugenics clause. In a letter to the *British Medical Journal*, they noted Sweden for permitting abortion ‘where it may reasonably be assumed that the mother or father of the expected child will, owing to hereditary disposition, transmit to their offspring, insanity, mental deficiency, or serious physical disease’. Moreover, the Swedish law stipulated abortion could not be performed in such cases ‘unless the woman is also sterilised, except when the operation cannot be performed owing to the woman lacking the power to give valid consent, or, for special reasons, it is found to be undesirable’. They pointed out that in 1946 the Swedish law was amended to broaden the eugenic indications so that abortion was permitted ‘whenever it is anticipated that the child by inheritance will be insane, imbecilic, or seriously handicapped by sickness or malformation’. They concluded, ‘we believe it is urgently necessary in this country to widen the range of indications for legal termination of pregnancy’.^106^

Browne, unsurprisingly, disagreed with the new direction ALRA was taking. She believed a new abortion law should be much simpler – and simply feminist. A new law, she said, should have

… a brief yet comprehensive formula such as: ‘Nothing in any Act shall be held to prejudice the right of a woman to ask a physician to terminate a pregnancy which is unwelcome to her, or the right of a physician to terminate such a pregnancy’. It is not ALRA’s business – in my humble view – to raise objections and suggest limitations to the right of maternal choice. Other persons and organisations will do that soon enough.^107^

Her intervention was ignored.

ALRA’s first chance to introduce new abortion legislation in Parliament was in 1952 when Labour MP Joseph Reeves offered to introduce a private member’s bill. When he asked for help in crafting a bill, Jenkins requested the assistance of a member of ALRA’s Medico-Legal Committee, Glanville Williams (1911–1997), a highly respected legal scholar; at the time he held a prestigious chair in jurisprudence at the University of London, and his reputation would grow immensely in subsequent decades.^108^ Williams immediately complied and the draft bill he crafted was a cautious document seeking only to reassure doctors they were legally permitted to perform abortions to save a woman’s life and her mental and physical health. It was quickly defeated in Parliament.

Asking Glanville Williams for help was both a telling and fateful decision, one that simultaneously reflected and would hugely reinforce ALRA’s growing emphasis on eugenics. Williams was an avowed eugenist who was alarmed by the supposedly declining quality of Britain’s genetic stock. In his much-admired book *Sanctity of Life and the Criminal Law* (1957) he claimed, ‘the fact of “recruitment from the bottom” in present society is beyond question’ and it was causing ‘the national stock of favourable genes’ to shrink. This was leading to a national decline in intelligence and an increase in the percentage of ‘feeble-minded children’. He believed that ‘by keeping alive mentally and physically ill-equipped children, we are opposing natural selection … [and] unless steps are taken to counteract this tendency, we shall as a race become progressively less fit.’ It was a ‘fact,’ he wrote, ‘that the community is burdened with an enormous number of unfit members …’. Of all the grounds upon which abortion should be granted, therefore, the ‘strongest’ one had nothing to do with women’s wellbeing: it was eugenic:

The strongest case is undoubtedly where the child is likely to suffer from a serious defect, either because of inheritance from one or both of his parents or because of some disease contracted by his mother during pregnancy. To allow the breeding of defectives is a horrible evil, far worse than any that may be found in abortion.

He regretted that pregnant ‘feebleminded’ women were unable to understand doctors’ explanations of the risk of having a damaged child, ‘in which case her child must be allowed to be born’.^109^

Like Chance and Jenkins, Williams was impressed with the Swedish abortion law that broke so thoroughly from ‘traditional notions’ about abortion. He, too, advocated eugenic abortion followed by sterilisation for ‘defectives’ who ‘are prone to sexual irresponsibility’. It was another ‘fact’ that ‘circumstances do sometimes impose upon the authorities of an institution the unhappy necessity of saying to an inmate [who is defective] that she either must be refused discharge or be sterilised’.

Eugenic abortion is resisted on the ground that this work should be done not by abortion but by contraception. Yet contraception is useless for people, such as mental defectives, who are unable or unwilling to practise it. There are many defectives who are not thought to require institutional treatment, who yet are prone to sexual irresponsibility; for these, abortion accompanied by sterilisation is a socially desirable operation.

He declared:

Sterilisation settles the problem once and for all … The obvious social importance of preventing the birth of children who are congenitally deaf, blind, paralysed, deformed, feeble-minded, mentally diseased, or subject to other serious hereditary afflictions, and the inadequacy of contraception for this purpose, has naturally given rise to the proposal to use sterilisation of the unfit as a means of racial improvement.^110^

Over all, Williams lamented that the British government failed ‘to realise the social importance’ of eugenics because of ‘the pressure of democratic opinion which refuses to see any genetic differences, even in terms of the broadest tendencies, between the different social classes’. For this reason he admired the United States, ‘the pioneer’ country that first acknowledged ‘the writings of Sir Francis Galton’ and, among other things, passed sterilization laws ‘as a measure of negative eugenics …’ .^111^

In 1958, Williams accepted the Eugenics Society’s invitation to become a member and within months was elected a Fellow. ALRA welcomed Williams’s ideas about negative eugenics, in fact they elected him president in 1956 and in 1958 they offered to promote his book.^112^ He remained their trusted legal mentor until 1967 and the author of all ALRA’s subsequent drafts of abortion bills.

Soon after the Reeves bill was defeated, Jenkins reached out to the Eugenics Society, implying she had a legitimate claim, ‘as a Fellow … for over twenty years’, for support for the draft bill going forward.^113^ But the Society was reluctant because ‘the proposed amendment did not go far enough in that it did not include any reference to the risk that the child might be born in some way gravely handicapped’. Instead, they complained, it referred only to women. However, they took up Jenkins’s suggestion that the two groups meet, which they did in November 1953 when the Society proposed adding a eugenics clause.^114^ Days later, Williams produced a new draft bill that advocated legalising abortion on two grounds, the first reflecting ALRA’s consistent concern for women, the second the Society’s (and Williams’s) negative eugenics:

- for the purpose of preventing injury to the mother in body or health;
- in the belief that there was grave risk of the child being born grossly deformed or lacking normal physical or mental faculties or incapable of normal physical or mental development.^115^

In January 1954, the Eugenics Society’s Council unanimously endorsed the revised bill.^116^

Also in 1954, Lord Amulree offered to champion abortion law reform, and Williams asked Jenkins, ‘I wonder is there any chance of persuading him to adopt the somewhat wider form [of the bill] that we agreed with the Eugenics Society?’^117^ Jenkins asked, but Amulree withdrew his support for reasons unclear.^118^ While looking for another MP to take up their cause, an ALRA member sponsored a motion at the annual meeting of the London Magistrates’ Association seeking to protect doctors who procured abortions to save a woman’s life or ‘if the child was to be mentally or physically incapable of normal development’, which passed.^119^

After meeting with the Eugenics Society in 1953, Jenkins and Williams made abortion on grounds of foetal ‘defect’ – what they themselves repeatedly called eugenic abortion – central to the campaign. Their collaboration became especially close after Jenkins lost her two comrades: Janet Chance committed suicide in 1953 and, two years later, Stella Browne died, taking the remnants of ALRA’s radical feminist spirit with her. Jenkins’s increased preoccupation with negative eugenics is evident in her own book *Law for the Rich* (1960) in which she emphatically restates her outrage at the harm working-class women suffer because of restrictive abortion laws – the title itself is a feminist slogan from the 1930s. But she also endorsed abortion for ‘damaged’ embryos. She explained she wished to prevent suffering caused by disability; as a mother of three, ‘love of children and hatred of seeing them suffer are amongst my strongest characteristics’. Then she added, ‘if carried to term and born alive’, a damaged embryo was ‘fated to need special treatment, often at public expense, throughout its life’, and stated eugenic abortion should be allowed ‘in view of our large numbers of mental defectives and infants born with other defects and congenital abnormalities’. Jenkins was clear that ALRA opposed *mandatory* abortion, nevertheless, she went on to once again endorse Sweden’s abortion law that made mandatory sterilisation a condition of receiving an abortion for some women and praised the ‘moral courage’ of a medical officer of health who insisted that a husband and wife ‘of six or seven children’ be sterilised. The procedure, she wrote, was not castration as many ‘unenlightened’ people believed; therefore, it would not affect a couple’s sexual relationship.^120^ In his introduction to Jenkins’ book, Williams deplored that ‘Neither Government nor Parliament has attempted even a start upon the most important task of all – the improvement of our eugenic heritage’.^121^

In the late 1950s, Jenkins was encouraged by growing public interest in abortion law reform resulting from increased medical knowledge about ways a foetus could be damaged in utero. New technologies for identifying foetal impairment developed in the 1950s coupled with preexisting fear and hostility to disability, led to increased popular acceptance of abortion. As a doctor explained in the *British Medical Journal* in 1960: ‘Today the main interest in what might be regarded as eugenic aspects of therapeutic abortion is not concerned with inherited mental disorders so much as with foetal injury and disease resulting from adverse agents operating during early intrauterine life’. He cited as examples exposure to excessive doses of X-rays and some viral infections, especially rubella.^122^ Heartened, ALRA in 1958 again amended its draft abortion legislation, this time along the lines of Sweden’s. After consulting the Eugenics Society, Williams drafted a broader eugenics clause permitting abortion ‘when there was grave risk of the child being born grossly deformed or with a physical or mental abnormality which would be of a degree to require constant hospital treatment or special care throughout life’.^123^ Reflecting the campaign’s saturation in eugenics and medicalisation of abortion by this point, ALRA renamed their proposed law the Medical Abortion Bill.^124^

On 10 February 1961, a Labour MP, Kenneth Robinson, offered to introduce the bill, now called the Medical Termination of Pregnancy Bill, as a private member’s bill. He supported the expanded eugenics clause, though he admitted in Parliament he had struggled with the decision, indicating the issue’s controversiality.^125^ He said, ‘if a woman suffers from German measles in the first month of pregnancy and is faced with a risk of up to 85 percent that her child will be seriously deformed or mentally defective, and asks that the pregnancy be terminated, the doctor should be permitted by the law to exercise his unfettered judgement in the matter’.^126^ The British Medical Association (BMA) itself had endorsed eugenic abortion as early as the 1930s.^127^ But the bill failed.

And then, in the early 1960s, there was the thalidomide catastrophe that, as explained above, greatly smoothed the path towards law reform. Members of ALRA wrote about the ‘profound sense of shock and horror’ the thalidomide disaster produced and were unequivocal about its importance to their success in achieving abortion law reform.^128^ After the effects of thalidomide were publicised a national poll reported 72 percent of the British public was in favour of abortion ‘where a child might be born deformed’.^129^ In addition to changing perceptions of abortion, thalidomide reanimated ALRA organisationally as soaring numbers of younger women, angry at the inaccessibility of abortion to terminate foetuses potentially damaged by the harmful drug, joined and a new leadership emerged.^130^ In the words of Madeleine Simms, a leader among the new generation of members: ‘When a woman is confronted with a medical diagnosis showing that she may give birth to a severely handicapped child, or still worse to a monster, she knows she has a problem that could be with her for a lifetime’.^131^ (In 1970, Simms was appointed Research Fellow at the Eugenics Society.^132^) Simms explains that already by 1963 public opposition to abortion was fading and that it was ‘above all the unforgettable experience of thalidomide’ responsible.^133^

Now supportable as a public health necessity, the bill began moving through the legislative process despite ongoing fierce opposition from Catholic MPs. Its success was all but assured once the bill won the support of the medical profession. This had not been easy. Significantly, the BMA supported the eugenics clause; it was the attempt to greatly increase women’s access to abortion they opposed. ALRA had to fight to have threats to women’s health and wellbeing accepted as indications for legal abortion and to do so they compromised, agreeing to the more limited wording in what became section 1(a) of the legislation. Finally, the bill passed; events extensively analysed elsewhere.

When the bill passed in October 1967, ALRA’s euphoria was more than a little tainted by their bitter disappointment that so much feminist ground had been lost to medical interests.^134^ The Abortion Act 1967 did not give women the right to procure abortion, nor was it ever meant to. Rather than focussing on women’s needs, the Act was intentionally designed to shield doctors from legal difficulty and secure their control over the process of determining who should be permitted to have abortions.^135^ Ultimately, however, the outcome was the logical conclusion to the previous twenty years of ALRA’s campaign, in which they deliberately downplayed their feminist agenda and embraced the medicalisation of abortion and negative eugenics.

## Conclusion

This essay demonstrated ALRA’s leading role in codifying eugenic abortion in the Abortion Act 1967. Throughout its existence, ALRA sought to expand access to safe abortion for the sake of working-class women’s health and wellbeing. However, in the decades after 1945, a period of intense social conservatism, they subordinated their feminism in order to win allies for abortion law reform in the male-dominated medical profession and eugenics movement. The one idea on which feminists, doctors and eugenists could all agree was that abortion on grounds of foetal impairment was socially desirable and morally defensible. Therefore, in the early 1950s ALRA adopted and merged the socially comprehensible discourses on medicalisation and eugenics in a revised campaign that now advocated two reasons for reforming the law: to protect women’s health and wellbeing and when there is indication of foetal impairment. As in other national contexts, the demand for access to abortion to prevent the birth of a disabled child was more persuasive than feminist claims to the right to reproductive control in winning popular and official support for abortion law reform in Britain. Tracing their campaign from the 1930s through to the 1960s sheds further light on the continuing power of eugenic thinking post-1945.

This essay furthers our understanding of feminist complicity in promoting a discriminatory discourse about disability in the postwar era. While doing so was plainly effective in campaigns to *expand* access to abortion, the reproductive rights movement is now, ironically, facing attacks fueled by their success at deploying disability.^136^ Today anti-abortionists in Britain, Europe and the United States are aggressively instrumentalising disability in their relentless effort to *undermine* women’s social autonomy. Arguing there is an insuperable conflict between disability rights and reproductive rights, they assert, falsely, that abortion on grounds on foetal impairment is inherently discriminatory towards disabled people and therefore should be prohibited by law. Worryingly, this tactic is proving effective for their cause. In Germany in 2009, for example, they succeeded in amending federal abortion legislation to intensify restrictions on abortions on grounds of foetal impairment. In the United States, since 2013 at least four states – Indiana, Ohio, Missouri and North Dakota – have introduced or passed legislation criminalising abortions sought for the same reason. And in 2020, the Polish Constitutional Court ruled that abortion on grounds of foetal impairment was unconstitutional, decreeing it contrary to the protection of the dignity and the life of the human person.^137^ As Dagmar Herzog asserts, the insensitive invocation of disability in the pro-choice rhetoric of the 1960s and 1970s has come to ‘haunt the abortion politics of the twenty-first century’.^138^

Disability rights advocates deplore this exploitation of their hard-won gains of the past two decades.^139^ Kendall Ciesemier, who was born with life-threatening disabilities, sums up the perspective of many when she expresses disgust at how ‘[a]bortion opponents like to use disabled fetuses as pawns to support their politics … By invoking a story about valuing disability’, she writes, ‘abortion opponents can connect abortion to the dark practice of eugenics …’.^140^ International organisations dedicated to advancing both reproductive rights and disability rights are also alarmed. In 2018 the UN Committee on the Elimination of Discrimination Against Women and the Committee on the Rights of Persons with Disabilities issued a joint statement condemning those who justify restricting women’s access to abortion by referring to disability rights, calling this ‘one of the most pressing issues that affects women and girls’. The leaders of the two committees reiterate that disability rights and gender equality are both components of the same human rights standard that should not be construed as conflicting. Regression on respect for reproductive rights threatens all women, they assert, including disabled women.^141^

Feminists rightfully criticise the demand of anti-abortionists that prospective parents ‘embrace disability parenthood’, calling it both ‘morally presumptuous and unattuned’ to parents’ possible vulnerabilities, such as socioeconomic or emotional precarity.^142^ At the same time, there is a need for the reproductive rights movement to confront ongoing discrimination against disabled people. Michelle Jarman writes we must ‘advocate for the value of disabled lives’ and we must do so by demanding ‘political and social structures of support for people with disabilities – beyond the womb’.^143^ Unless and until such structures exist, the charge that abortion on grounds of foetal impairment is discriminatory ‘carries some weight’.^144^ In sum, it is important to heed the call of disability rights activists and scholars to respectfully engage with the politics of disability.^145^ As Alison Piepmeier contends, ‘We need scholarly and activist feminist conversations about reproduction that embrace, rather than fear, the complexity of reproductive decision making’.^146^ Such conversations require a fuller understanding of feminism’s problematic history of promoting discriminatory discourses about disabled people, a tendency that some disability rights activists argue has ‘persisted or go[es] unchallenged in the reproductive rights movement today.’^147^ Confronting feminism’s entanglement with eugenics will not just enrich the historiographies on feminism, the long eugenics and abortion. It will also enhance discussions among feminists needing to learn from our past and between feminists and disability rights activists who need each other in the ongoing fight for reproductive and sexual freedom.

## Footnotes

1

Alison Bashford , ‘Epilogue: Where Did Eugenics Go?’, in Alison Bashford and Philippa Levine (eds.), The Oxford Handbook of the History of Eugenics (Oxford: Oxford University Press, 2010), 539–58; 539.

2

Alison Bashford and Philippa Levine , ‘Introduction’, in Alison Bashford and Philippa Levine (eds.), The Oxford Handbook of the History of Eugenics (Oxford: Oxford University Press, 2010), 2–24 ; 3–4. Important interventions include: Troy Duster , Backdoor to Eugenics (London and New York: Routledge, 1990); Diane Paul , The Politics of Heredity: Essays on Eugenics, Biomedicine, and the Nature-Nurture Debate (Albany: State University of New York Press, 1998); Frank Dikötter , Imperfect Conceptions: Medical Knowledge, Birth Defects, and Eugenics (New York: Columbia University Press, 1998); Carolyn Burdett , ‘Introduction: Eugenics Old and New’, New Formations, 60 (2007), 7–12 ; Merryn Ekberg , ‘The Old Eugenics and the New Genetics Compared’, Social History of Medicine, 20, 3 (2007), 581–93; Diane Paul , ‘On Drawing Lessons from the History of Eugenics,’ in Lori P. Knowles and Gregory E. Kaebnick (eds.), Reprogenetics: Law, Policy, and Ethical Issues (Baltimore: Johns Hopkins University Press, 2007), 3–19 .

3 Bashford, *op. cit.* (note 1), 551.

4

Robert G. Resta , ‘Eugenic Considerations in the Theory and Practice of Genetic Counseling’, Science in Context, 11, 3–4 (1998), 431–3815168672 10.1017/s0269889700003124; Ruth Hubbard , ‘Abortion and Disability: Who Should and Should Not Inhabit the World?’, in Lennard J. Davis (ed.), The Disability Studies Reader (New York: Routledge, 2010), 74–86 ; Michael J. Sandel , The Case against Perfection: Ethics in the Age of Genetic Engineering (Cambridge, MA: Belknap Press, 2007).

5 Bashford, *op. cit.* (note 1), 542.

6 In this essay, I define disability as a ‘culturally fabricated narrative’ that devalues people whose bodies and intellectual capacities do not conform to normative cultural standards. Rosemarie Garland-Thomson, ‘Integrating Disability, Transforming Feminist Theory’, *Feminist Formations*, 14, 3 (2002), 1–32; 5.

7

Astghik Hovhannisyan , ‘Preventing the Birth of “Inferior Offspring”: Eugenic Sterilizations in Postwar Japan’, Japan Forum, 33, 3 (2021), 383–401; 386.

8

Joanna Schoen , Choice and Coercion: Birth Control, Sterilization, and Abortion in Public Health and Welfare (Chapel Hill: University of North Carolina Press, 2005), 143 ; Mary Ziegler , ‘The Disability Politics of Abortion’, Utah Law Review, 3 (2017), 586–631; 595.

9

Susanne M. Klausen , ‘“There is a Row about Foetal Abnormality Underway”: The Debate about Inclusion of a Eugenics Clause in the Contraception, Sterilisation, and Abortion Act, 1977–1978’, New Zealand Journal of History, 51, 2 (2017), 80–103 .

10

Dagmar Herzog , Unlearning Eugenics: Sexuality, Reproduction, and Disability in Post-War Nazi Europe (Madison: University of Wisconsin Press, 2018), 9.

11

Dagmar Herzog , ‘Abortion, Christianity, Disability: Western Europe, 1960s–1970s’, Archiv für Sozialgeschichte, 51 (2011), 375–400 ; 399.

12 In this paper, I follow the social model of disability’s view that people with mental and physical impairments are *disabled* by social restrictions that prevent their ability to participate in society on an equal basis with the abled. Accordingly, disability is political. It is a matter of social justice that barriers to equal participation in society be dismantled. The social model of disability emerged out of the disability rights movement starting in the 1970s. For an introduction to the model and its history, see Tanya Burchardt , ‘Capabilities and Disability: The Capabilities Framework and the Social Model of Disability’, Disability and Society, 19, 7 (2004), 735–51 and Mike Oliver , ‘The Social Model of Disability: Thirty Years On’, Disability and Society, 28, 7 (2013): 1024–6.

13

Sharon L. Snyder and David T. Mitchell , Cultural Locations of Disability (Chicago: University of Chicago Press, 2006).

14 Bashford, *op. cit.* (note 1), 546.

15 Section 58 of the Offences Against the Person Act 1861 states: ‘Every Woman, being with Child, who, with Intent to procure her own Miscarriage, shall unlawfully administer to herself any Poison or other noxious Thing, or shall unlawfully use any Instrument or other Means whatsoever with the like Intent, and whosoever, with Intent to procure the Miscarriage of any Woman, whether she be or be not with Child, shall unlawfully administer to her or cause to be taken by her any Poison or other noxious Thing, or shall unlawfully use any Instrument or other Means whatsoever with the like Intent, shall be guilty of Felony, and being convicted thereof shall be liable, at the Discretion of the Court, to be kept in Penal Servitude for Life or for any Term not less than Three Years – or to be imprisoned for any Term not exceeding Two Years, with or without Hard Labour, and with or without Solitary Confinement.’ Section 59 states: ‘Whosoever shall unlawfully supply or procure any Poison or other noxious Thing, or any Instrument or Thing whatsoever, knowing that the same is intended to be unlawfully used or employed with Intent to procure the Miscarriage of any Woman, whether she be or be not with Child, shall be guilty of a Misdemeanor, and being convicted thereof shall be liable, at the Discretion of the Court, to be kept in Penal Servitude for the Term of Three Years, or to be imprisoned for any Term not exceeding Two Years, with or without Hard Labour.’ https://www.legislation.gov.uk/ukpga/Vict/24-25/100/section/59/enacted. Section 1(1) of the Infant Life Preservation Act 1929 states that other than abortions performed to save a woman’s life, ‘any person who, with intent to destroy the life of a child capable of being born alive, by any wilful act causes a child to die before it has an existence independent of its mother, shall be guilty of felony, to wit, of child destruction, and shall be liable on conviction thereof on indictment to penal servitude for life.’ https://www.legislation.gov.uk/ukpga/Geo5/19-20/34/section/1.

16 Abortion Act 1967, s 1(b).

17 A representative example is Rebecca Cook and Bernard M. Dickens, *Three Studies of Abortion Laws in the Commonwealth* (London: Commonwealth Secretariat, 1977), table 1. Cook and Dickens, both highly respected legal scholars and advocates of accessible abortion, use the term ‘eugenic (fetal impediment)’ for an indication for abortion in their survey of abortion laws for the Commonwealth Secretariat.

18

Lucy Bland and Lesley A. Hall , ‘Eugenics in Britain: The View from the Metropole’, in Alison Bashford and Philippa Levine (eds.), The Oxford Handbook of the History of Eugenics (Oxford: Oxford University Press, 2010), 212–27; 223.

19 For analysis of attempts to amend the Act, see Sally Sheldon et al., The Abortion Act 1967: A Biography of a UK Law (Cambridge, UK: Cambridge University Press, 2022). In 2020, three claimants took the British government to court to overthrow s 1(b) of the Act. They were Heidi Crowter, who lives with Down’s syndrome, and Maire Lea-Wilson, who is the mother of the third claimant, A, a two year old with Down’s syndrome. They argued the legality of terminating a foetus on grounds of foetal impairment up to the point of birth is discriminatory. In October 2021, the High Court dismissed their case. Zoe L. Tongue , ‘
*Crowter v. Secretary of State for Health and Social Care*: Discrimination, Disability, and Access to Abortion’, Medical Law Review, 30, 1 (2022), 177–8734850054 10.1093/medlaw/fwab045.

20

Keith Hindell and Madeleine Simms , Abortion Law Reformed (London: Peter Owen Publishers, 1971), 87 ; 108–112; 117. For the importance of the thalidomide crisis to purging the association of abortion with promiscuity and criminality in other countries, see Clare Parker , ‘From Immorality to Public Health: Thalidomide and the Debate for Legal Abortion in Australia,’ Social History of Medicine, 25, 4 (2012), 863–80 and Susanne M. Klausen and Julie Parle , ‘“Are We Going to Stand By and Let These Children Come Into the World?”: The Impact of the “Thalidomide Disaster” in South Africa, 1960–1977’, Journal of Southern African Studies, 41, 4 (2015), 735–52.

21 Hindell and Simms, *ibid.*, 112.

22 In 1989, the Eugenics Society changed its name to the Galton Institute, which in 2021 changed its name to the Adelphi Genetics Forum.

23 The state of South Australia (1969); India (1971); Zambia (1972); Australia’s Northern Territory (1973); Jammu and Kashmir (1974) and Cypress (1974). Cook and Dickens, *op. cit.* (note 17), table 1.

24 Abortion and Sterilisation Act 1975, s 3 (1)(c) permitted abortion ‘where there exists a serious risk that the child to be born will suffer from a physical or mental defect of such a nature that he will be irreparably seriously handicapped, and two other medical practitioners have certified in writing that, in their opinion, there exists, on scientific grounds, such a risk’. Cited in Susanne M. Klausen , Abortion under Apartheid: Nationalism, Sexuality, and Women’s Reproductive Rights in South Africa (London and New York: Oxford University Press, 2015), 222 .

25 Namely in cases where ‘there is substantial risk that the child, if born, would be so physically or mentally abnormal as to be seriously handicapped.’ Contraception, Sterilisation and Abortion Act 1977, s 1(b), amended 1978, and Section 187A of the Crimes Act 1961.

26 United Nations, Department of Economic and Social Affairs, *Population Division, World Abortion Policies 2013*, http://www.un.org/en/development/desa/population/publications/pdf/policy/WorldAbortionPolicies2013/WorldAbortionPolicies2013_WallChart.pdf.

27

Barbara Brookes , Abortion in England, 1900–1967 (London: Croom Helm, 1988); Stephen Brooke , ‘“A New World for Women”? Abortion Law Reform in Britain during the 1930s’, American Historical Review, 106, 2 (2001), 431–5918186164 ; Stephen Brooke , Sexual Politics: Sexuality, Family Planning, and the British Left from the 1880s to the Present Day (London and Oxford: Oxford University Press, 2011); Lesley A. Hall , The Life and Times of Stella Browne (London: I. B. Taurus, 2011); Emma L. Jones, ‘Attitudes to Abortion in the Era of Reform: Evidence from the Abortion Law Reform Association Correspondence’, *Women’s History Review*, 20, 2 (2011), 283–98.

28

Kate Gleeson , ‘Persuading Parliament: Abortion Law Reform in the UK’, Australasian Parliamentary Review, 22, 2 (2007), 23–42; 28.

29

Ann Farmer , By Their Fruits: Eugenics, Population Control, and the Abortion Campaign (Washington, DC: The Catholic University of America Press, 2008).

30 Farmer, *ibid.*, xiii, 358.

31 Farmer, *ibid.*, 88.

32 Farmer, *ibid.*, 66; 84.

33 Farmer, *ibid.*, xii.

34 For studies of the latter, see Paul Weindling , ‘International Eugenics: Swedish Sterilization in Context’, Scandinavian Journal of History, 24, 2 (1999), 179–9722256389 10.1080/03468759950115791; Stefan Kühl , The Nazi Connection: Eugenics, American Racism and German National Socialism (Oxford and New York: Oxford University Press, 1994); Nils Roll-Hansen , ‘Scandinavian Eugenics in the International Context’, in Gunnar Broberg and Nils Roll-Hansen (eds.), Eugenics and the Welfare State: Sterilization Policy in Denmark, Sweden, Norway, and Finland (East Lansing, MI: Michigan State University Press, 1996); David T. Mitchell and Sharon Snyder , ‘The Eugenic Atlantic: Race, Disability, and the Making of an International Eugenic Science, 1800–1945’, Disability and Society, 18, 7 (2003), 843–86; Ian Dowbiggin , The Sterilization Movement and Global Fertility in the Twentieth Century (New York: Oxford University Press, 2008); Alison Bashford , ‘Internationalism, Cosmopolitanism, Eugenics’, in Alison Bashford and Philippa Levine (eds.), The Oxford Handbook of the History of Eugenics (Oxford: Oxford University Press, 2010), 154–72.

35 See notes on abortion laws in nineteen other countries in Archives and Manuscripts, Wellcome Library, London, Records of the Abortion Law Reform Association, 1935–1970 (hereafter ALRA Papers), SA/ALR/A.12/1: ALRA: Legislation, Statistics, Information; Abortion Laws in Other Countries, 1932–1970.

36 In September 1936, Browne told the ALRA executive committee, of which she was a member, that she was interested in forming an ALRA library ‘to contain copies of foreign abortion laws in addition to books and pamphlets’, which she did within a month. In October ‘the committee asked Miss Browne to prepare a pamphlet on foreign aspects of abortion laws’. Minutes of Executive Committee, 21 September 1936 and 19 October 1936, ALRA Papers, SA/ALR/A.1/2/1, Executive Committee and Annual General Meetings (hereafter ALRA EC Minutes).

37

F. W. Stella Browne , ‘The Right to Abortion’, in F. W. Stella Browne , Anthony Mario Ludovici and Harry Roberts (eds.), *Abortion* (London: George Allen and Unwin, 1935), 13–50 ; 21. For a contemporary example of a reproductive rights group in another national context influenced by transnational feminist actions, see Maud Bracke , ‘Our Bodies, Ourselves: The Transnational Connections of 1970s Italian and Roman Feminism’, Journal of Contemporary History, 50, 3 (2015), 560–80.

38 The Act had no residency requirements, so once the law was enacted women from around the world started travelling to London in search of medical abortionists. Christabelle Sethna , ‘All Aboard? Canadian Women’s Abortion Tourism, 1960–1980’, in Cheryl Krasnik Warsh (ed.), Women’s Health History in North America (Waterloo: Wilfred Laurier University Press, 2011), 89–108 ; Christabelle Sethna and Marion Doull , ‘Journeys of Choice? Abortion, Travel, and Women’s Autonomy’, in Stuart J. Murray and Dave Holmes (eds.), Critical Interventions in the Ethics of Healthcare: Challenging the Principle of Autonomy in Bioethics (Surrey, England: Ashgate Publishers, 2009), 163–79; Klausen, *op. cit.* (note 24); and Gayle David and Christabelle Sethna (eds.), Abortion across Borders: Transnational Travel and Access to Abortion Services (Baltimore, Maryland: Johns Hopkins University Press, 2019).

39 As ALRA’s secretary Diane Munday explained in 1971 to a British woman trying to help a group of pregnant French students come to London: “I am not able to give you any information regarding procedures for helping foreign women to obtain abortions in Britain. Although the Law contains no residency qualification, and although during our campaign for a more liberal law none of us were thinking only of British women, the political situation has become such since the Abortion Act came into force that we have to do all in our power to safeguard the Act as it stands, not only for the immediate benefit of British women but for the long-term benefit of people from overseas who are now looking to us for an example as they discuss the change of their own laws.” Diane Munday to Juliet Gowen, London, 17 May 1971, ALRA Papers, SA/ALR/H.30, Abortion: Other Countries, c. 1965–1977, Box E–H.

40 For example, in 1970 an Australian woman wrote to Mundy: “I will be returning to Sydney … and would very much like to contact the Australian branch of your association. I have a great deal of admiration for the work you have done here in England and if possible I would like to help bring about such reforms in my country. Do you have any literature which would be of help to me? I would appreciate very much hearing from you, and if you put me in touch with a contact in Australia I would be most grateful.” Two days later, Munday sent a reply: ‘Thank you for your letter … Enclosed is some current material about the Abortion Law Reform association which I hope will be of interest and use to you. We are, of course, in contact with the various Abortion Law Reform Associations in Australia and the following names and addresses may be of interest to you ….’ Valerie Tobin to Diane Munday, London, 23 March 1970, and Mundy to Tobin, 25 March 1970, ALRA Papers, SA/ALR/H.29, Abortion: Other Countries, c. 1965–1977, Box A–D.

41

Donald Mackenzie , ‘Eugenics in Britain’, Social Studies of Science, 6 (1976) 499–532 11610196 10.1177/030631277600600310; Bland and Hall, *op. cit.* (note 18); Paul Weindling, ‘Julian Huxley and the Continuity of Eugenics in Twentieth-century Britain’, *Journal of Modern European History / Zeitschrift für moderne europäische Geschichte / Revue d’histoire européenne contemporaine*, 10, 4 (2012), 480–99; Alex Aylward , ‘R. A. Fisher, Eugenics, and the Campaign for Family Allowances in Interwar Britain’, The British Journal for the History of Science, 54 (2021), 485–505 34629128 10.1017/S0007087421000674.

42 Mackenize, *ibid.*, 501, 510.

43 Mitchell and Snyder, *op. cit.* (note 34), 845.

44 Tony Siebers, *Disability Theory* (Ann Arbor: University of Michigan Press, 2008); Garland-Thompson, *op. cit.* (note 6); Rosemarie Garland-Thompson, ‘Building a World with Disability in It’, in Anne Waldschmidt, Hanjo Berressem and Moritz Ingwersen (eds.), *Culture – Theory – Disability: Encounters between Disability Studies and Cultural Studies* (transcript Verlag, 2017), 51–62; Gesine Wegner , ‘“[L]ess than accessible and seriously disheartening”: Unfolding Transatlantic Eugenics in Disability Scholarship’, Amerikastudien / American Studies, 64, 2 (2019), 189–214 .

45

Michael Rembis , ‘Disability and the History of Eugenics’, in Michael Rembis , Catherine Kudlick and Kim E. Nielsen (eds.), The Oxford Handbook of Disability History (New York: Oxford University Press, 2018), 85–104 ; 86–87.

46 Brookes, *op. cit.* (note 27).

47 ALRA Newsletter, 24 January 1937, ALRA Papers, SA/ALR/A.2/1, Pre-1949 material: 1930–1937 (hereafter ALRA Newsletter).

48 Sheelagh McGuiness, ‘Law, Reproduction, and Disability: Fatally “Handicapped”?’, *Medical Law Review*, 21, 2 (2013), 213–242. The Infant Life (Preservation) Act 1929 amended the Offences Against the Person Act 1861 by making it legally permissible for a doctor to perform an abortion if it was performed ‘in good faith for the purpose only of preserving the life of the mother’, https://www.legislation.gov.uk/ukpga/Geo5/19-20/34/section/1/enacted.

49 Brookes, *op. cit.* (note 27); Stephen Brooke, ‘Jenkins [nee Glyde], Alice Brook (1886–1967)’, in *Oxford Dictionary of National Biography* (Oxford and New York: Oxford University Press, 2004), online edn., https://www.oxforddnbcom.proxy.library.carleton.ca/search?q=alice+jenkins&searchBtn=Search&isQuickSearch=true.

50 Lesley A. Hall, ‘“I Have Never Met the Normal Woman”: Stella Browne and the Politics of Womanhood’, *Women’s History Review*, 6, 2 (1997), 157–82; 161.

51 F. W. Stella Browne, ‘The Feminine Aspect of Birth Control’, in R. Pierpoint (ed.), *Report of the Fifth International Neo-Malthusian and Birth Control Conference* (London: William Heinemann, 1922), 40–44, cited in Hall, ‘“I Have Never Met the Normal Woman,”’ 158.

52 See, for example, Janet Chance , The Cost of English Morals (London: Noel Douglas, 1932); Janet Chance , ‘Reform of the Sex Laws’, in C. E. M. Joad (ed.), Manifesto: Being the Book of the Federation of Progressive Societies and Individuals (London: George Allen and Unwin, 1934), 165–82.

53 Cited in Brookes, *op. cit.* (note 27), 90.

54 F. W. Stella Browne, *The Sexual Variety and Variability among Women and Their Bearing upon Social Re-construction* (London: C.W. Beaumont, 1917), 3.

55 Alice Jenkins, ‘Abortion and Maternal Mortality’, c. 1936, 1–6, ALRA Papers, SA/ALR/A.2/1, Pre-1949 material: 1930–1937. Above the title, Jenkins wrote ‘First address on this subject, at Liverpool with Mrs. Chance’.

56 Brooke, *op. cit.* (note 27), 102–3.

57 Brookes, *op. cit.* (note 27), 79–104. On the memberships and affiliations see ALRA, ‘Memorandum for Presentation to the Inter-Departmental Committee on Abortion from the Abortion Law Reform Association’, 1937, 1 (hereafter ALRA Memorandum), British National Archives, Richmond, UK, (hereafter The National Archives), MH 71/21.

58 ALRA Newsletter, October 1937, 1, ALRA Papers, SA/ALR/A.2/1, Pre-1949 material: 1930–1937.

59 ALRA Memorandum.

60 Minutes of Evidence at Eighth Meeting of the Interdepartmental Committee on Abortion, Ministry of Health, 17 November 1937: Evidence of Miss F. W. Stella Browne, The National Archives, MH71/23.

61 Browne, *op. cit.* (note 37), 31.

62 Janet Chance, ‘Chairman’s Statement on the Policy and Representation of A.L.R.A’, 1937, 1–2, ALRA Papers, SA/ALR/A.2/1, Pre-1949 material: 1930–1937.

63 Janet Chance, ‘ALRA: Notes for Speakers’, April 1937, ALRA Papers, SA/ALR/A.2/1, Pre-1949 material: 1930–1937.

64 Some examples of their activities: in 1938, Browne and Jenkins spoke at a ‘tea-time meeting’ attended by women at the Labour Women’s Conference; a Mrs. Saran spoke to a meeting of delegates at the Women’s Co-operative Guild Congress in Southampton; ALRA co-founder Dr. Joan Malleson and Browne spoke at a conference organised by the New Fabian Research Bureau Conference, where Browne had ‘put the case for radical reform’; and ALRA held a training session for members interested in giving talks to organisations. In 1939, Browne addressed an ALRA conference that was attended by delegates from twenty-five local labour and cooperative organisations and where attendees unanimously passed a ‘strong resolution’ that was sent to the Parliamentary Committee of the Labour Party; Chance addressed a group of sixty women in Scotland ‘representing several Women’s Cooperative Guilds and Labour Women’s Sections’ of Perth and Dundee, and another meeting of 250 people organised by the Rationalist Press Association; Browne addressed the Chichester Labour Women’s Section and delegates from ‘neighbouring Labour and Cooperative groups’, where a resolution was passed eighteen to one ‘in spite of eloquent Catholic opposition’; and other members addressed meetings organised by the Women’s Adults Schools, additional Labour Women’s Sections, the Left Book Club, the National Council of Women, the National Secular Society, the National Unemployed Workers Movement, Wives Fellowship, the Women’s Cooperative Guild and the Woodlanders’ Club.

65 ALRA Memorandum.

66 Hall, *op. cit.* (note 27), 233.

67 C.E.M. Joad, ‘The FPSI: What It Is; What It Wants; and How It Hopes to Obtain It’, in C.E.M. Joad (ed.), *Manifesto: Being the Book of the Federation of Progressive Societies and Individuals* (London: George Allen and Unwin,1934), 29–62; 30. See also letterhead of the WLSR, Archive for Sexology, https://web.archive.org/web/20110115073920/http://www2.huberlin.de/sexology/GESUND/ARCHIV/WLSR.HTM.

68

Greta Jones , ‘Women and Eugenics in Britain: The Case of Mary Scharlieb, Elizabeth Sloan Chesser, and Stella Browne’, Annals of Science, 51 (1995), 481–502 10.1080/0003379950020036111640067.

69

Ann Taylor Allen , ‘Feminism and Eugenics in Germany and Britain, 1900–1940: A Comparative Perspective’, German Studies Review, 23, 3 (2000), 477–505; 478. Studies on the formative role of eugenics in feminist birth-control campaigns include: Jane Carey, ‘White Anxieties and the Articulation of Race: The Women’s Movement and the Making of White Australia, 1910s–1930s’, in Jane Carey and Claire McLisky (eds.), Creating White Australia (Sydney: Sydney University Press, 2009), 195–213 ; Jane Carey , ‘The Racial Imperatives of Sex: Birth Control and Eugenics in Britain, the United States and Australia in the Interwar Years’, Women’s History Review, 21, 5 (2012), 733–52.

70

Mark B. Adams , The Wellborn Science: Eugenics in Germany, France, Brazil, and Russia (New York: Oxford University Press, 1990); Diane Paul , Controlling Human Heredity (New York: Humanity Books, 1995); Nancy Stepan , ‘The Hour of Eugenics’: Race, Gender, and Nation in Latin America (Ithaca, NY: Cornell University Press, 1992); Bashford and Levine, *op. cit.* (note 2), 2–24.

71 Lesley A. Hall, ‘Situating Stopes: or, Putting Marie in Her Proper Place’, keynote address at the IHR@90 event, 1990, published on Lesley Hall’s personal website, https://lesleyahall.blogspot.com/2012/02/website-update-situating-stopes.html.

72 Taylor Allen, *op. cit.* (note 69), 494.

73 Richard Soloway, *Demography and Degeneration: Eugenics and the Declining Birthrate in Twentieth-century Britain* (Chapel Hill, NC: University of North Carolina Press,1990), xviii.

74 Matthew Thomson, ‘Social Policy and the Management of the Problem of Mental Deficiency in Interwar London’, *The London Journal*, 18, 2 (1993), 129–42; Jan Walmsley, ‘Women and the Mental Deficiency Act of 1913: Citizenship, Sexuality and Regulation’, *British Journal of Learning Disabilities*, 18, 2 (2000), 45–87; Pamela Block, ‘Sexuality, Fertility, and Danger: Twentieth-Century Images of Women with Cognitive Disabilities’, *Sexuality and Disability*, 18, 4 (2000), 239–54; Pamela Dale, ‘Implementing the 1913 Mental Deficiency Act: Competing Priorities and Resource Restraint Evident in the South West of England before 1948’, *Social History of Medicine*, 16, 3 (2003), 403–18; Joyce Goodman, ‘Pedagogy and Sex: Mary Dendy (1855–1933), Feebleminded Girls and the Sandlebridge Schools, 1902–33’, *History of Education*, 34, 2 (2005), 171–87.

75 Mary Davis, *Sylvia Pankhurst: A Life in Radical Politics* (London: Pluto Press, 1999).

76 George Robb, ‘The Way of All Flesh: Degeneration, Eugenics, and the Gospel of Free Love’, *Journal of the History of Sexuality*, 6, 4 (1996), 589–603.

77 Thanks to Lesley A. Hall for bringing this to my attention.

78 F. W. Stella Brown, ‘A Few Straight Questions to the Eugenics Society’, *The Freewoman*, 2, 37 (1912), 217–18; 218.

79 Browne, *op. cit.* (note 54), 13–14.

80 Browne, *op. cit.* (note 78), 217; see also F. W. Stella Browne, ‘More Questions’, *The Freewoman*, 2, 39 (1912), 258.

81 Browne, *op. cit.* (note 54), 13–14.

82 Lesley A. Hall, ‘“Not a Domestic Utensil but a Woman and a Citizen”: Stella Browne on Women, Health and Society’, in Christopher Lawrence and Anna-K. Mayer (eds.), *Regenerating England: Science, Medicine and Culture in Inter-War Britain*, eds. (Amsterdam: Editions Rodopi, 2000), 275–302; 279.

83 See correspondence between Janet Chance and C. P. Blacker, Wellcome Trust Library and Archives, Eugenics Society, Archives and Manuscripts 1863–2008 (hereafter ES Papers), SA/EUG/C.65, Janet Chance, 1931–1951.

84 Soloway, *op. cit.* (note 73).

85 Minutes of ALRA Executive Committee (hereafter ALRA EC Minutes), 14 July 1944, ALRA Papers, SA/ALR/A.1/2/1, Executive Committee and Annual General Meetings.

86
*Ibid.*, 28 November 1945. On the Royal Commission on Population see http://discovery.nationalarchives.gov.uk/details/r/C13349.

87 Alice Jenkins, ‘Deterrents to Parenthood’, letter to the editor, *Eugenics Review,* 36, 1 (1944), 42. See also Alice Jenkins, letter to the editor, *Eugenics Review*, 35, 2 (1943), 48 and Alice Jenkins, letter to the editor, *Eugenics Review*, 35, 3–4 (1944), 96.

88 Stella Browne, ‘Report of Activities 1939–1945’, 1, ALRA Papers, SA/ALR/B.5, F. W. Stella Browne Correspondence.

89

Martin Pugh , ‘Domesticity and the Decline of Feminism, 1930–1950’, in Harold L. Smith (ed.), British Feminism in the Twentieth Century (Aldershot: Edward Elgar, 1990), 144–62.

90

Gavin Schaffer , *Racial Science and British Society, 1930*–*62* (Houndmills: Palgrave Macmillan, 2008).

91

Chris Waters , ‘“Dark Strangers” in Our Midst: Discourses of Race and Nation in Britain, 1947–1963’, Journal of British Studies, 36, 2 (1997), 207–38, 208.

92

Clare Hanson , Eugenics, Literature, and Culture in Post-war Britain (New York: Routledge, 2013), 149.

93 Bland and Hall, *op. cit.* (note 18), 223.

94

C. P. Blacker , Neurosis and the Mental Health Services (Oxford: Oxford University Press, 1948).

95 ALRA EC Minutes, 10 October 1945, 5, ALRA Papers, SA/ALR/A.1/2/1, Executive Committee and Annual General Meetings.

96

Alice Jenkins , Law for the Rich (London: Victor Gollancz, 1960), 59.

97 Brookes, *op. cit.* (note 24).

98 In 1938, eminent gynaecologist Dr. Aleck Bourne was prosecuted for performing an abortion on a fourteen-year-old girl who was gang raped by five soldiers and became pregnant as a result. He was charged with the offence of conducting an illegal abortion and acquitted. The presiding judge, Mr. Justice Macnaghten, stated in his judgment: ‘If the doctor is of the opinion, on reasonable grounds and with adequate knowledge, that the probable consequence of the continuance of the pregnancy will be to make the woman a physical or mental wreck, the jury are entitled to take the view that the doctor is operating for the purpose of preserving the life of the mother’. Thereafter it was acceptable for doctors to perform abortions if pregnancy was deemed a risk to the pregnant woman’s physical and mental health. See *Rex v. Bourne*, 3 All E. R. 615 (1938), available at https://www.law.utoronto.ca/sites/default/files/documents/reprohealth/united_kingdom_1938_bourne.pdf

99 ALRA EC Minutes, 23 March 1951, ALRA Papers, SA/ALR/A.1/2/1, Executive Committee and Annual General Meetings.

100 Alice Jenkins to Joseph Reeves, London, 26 November 1952, ALRA Papers, SAALR/A/3/1, Miscellaneous correspondence: 1949–1952.

101 Hanson, *op. cit.* (note 92), 121. The Eugenics Society helped create the International Planned Parenthood Association, established in 1952, in order to intervene in ‘excessive’ rates of population growth in what was becoming known as the Third World. Matthew Connelly , Fatal Misconception: The Struggle to Control World Population (Cambridge, Mass.: Belknap Press of Harvard University Press, 2008).

102 Alice Jenkins, ‘Is Briton Overpopulated?’, letter to the editor, *Eugenics Review*, 38, 2 (1946), 105–6; 106.

103 Minutes of ALRA Annual General Meeting, 28 September 1949, ALRA Papers, SA/ALR/A.1/2/1, Executive Committee and Annual General Meetings.

104 Janet Chance to Miss Schenk, 29 June 1950, ES Papers, SA/EUG/C.65, Janet Chance, 1931–1951.

105 Cited in Bashford, *op. cit.* (note 1), 546.

106

Janet Chance and Alice Jenkins , letter to the editor, ‘Legal Termination of Pregnancy’, British Medical Journal, 2, 4734 (1951), 796 .

107 Stella Browne to Janet Chance, 12 December 1951, ALRA Papers, SA/ALR/B.5, F. W. Stella Browne Correspondence.

108 ‘Glanville Llewellyn Williams, 1911–1997’, *Proceedings of the British Academy*, 115 (2002), 411–35.

109

Glanville Williams , The Sanctity of Life and the Criminal Law (New York: Knopf, 1957), 70–1; 81; 233–4; 176.

110 Williams, *ibid.*, 236–45; 88; 234; 80.

111 Williams, *ibid.*, 73; 82.

112 ALRA EC Minutes, 25 June 1958, ALRA Papers, SA/ALR/A.1/2/1, Executive Committee and Annual General Meetings.

113 Alice Jenkins to the Eugenics Society, 19 September 1953, ES Papers, SA/EUG/C.192, Mrs. W. J. (Alice) Jenkins, 1933–1961.

114 Eugenics Society to Alice Jenkins, 2 November 1953, ALRA Papers, SA/ALR/A.3/2, Miscellaneous correspondence, 1953.

115 Proposed Bill attached in letter from Glanville Williams to Alice Jenkins, 10 December 1954, ALRA Papers, SA/ALR/A.3/3, Miscellaneous correspondence, 1954.

116 Eugenics Society to Alice Jenkins, 29 January 1954, ALRA Papers, SA/ALR/A.3/3, Miscellaneous correspondence, 1954.

117 Glanville Williams to Alice Jenkins, 23 January 1954, ALRA Papers, SA/ALR/A.3/3, Miscellaneous correspondence, 1954.

118

Eugenics Society , ‘Notes of the Quarter’, Eugenics Review, 46, 2 (1954), 76–7.

119 Brookes, *op. cit.* (note 27), 148.

120 Jenkins, *op. cit.* (note 96 *)*, 77–8; 74–5; 79.

121 Glanville Williams, ‘Introduction’, *Law for the Rich*, 11–20, here 11.

122

T. N. A Jeffcoate , ‘Indications for Therapeutic Abortion’, British Medical Journal, 1, 5173 (1960), 585–6.10.1136/bmj.1.5173.581PMC196677114406992

123 Alice Jenkins to Eugenics Society, 6 January 1958, ES Papers, SA/EUG/C.192, Mrs. W. J. (Alice) Jenkins, 1933–1961.

124 This occurred at the 1958 annual general meeting.

125 Vera Houghton, a long time ALRA member, wrote at the time that, ‘as was expected’, including the clause on foetal abnormality was ‘controversial’. Vera Houghton, ‘Medical Termination of Pregnancy Bill’, *Eugenics Review*, 53, 2 (1961), 94.

126 Kenneth Robinson’s speech contained in ALRA Papers, SA/ALR/B.8–20, Madeleine Simms’s Papers.

127 In 1936, a committee of the BMA recommended doctors be permitted to perform abortions when there ‘is reasonable certainty that serious disease will be transmitted to the child’, such as hereditary blood disorders and mental illness. Cited in Ziegler, *op. cit.* (note 8), 592.

128

Keith Hindell and Madeleine Simms , ‘How the Abortion Lobby Worked’, The Political Quarterly, 39, 3 (1968), 269–82; 273.

129 Hindell and Simms, *ibid.*, 274.

130 Hindell and Simms, *ibid.*; Keith Hindell and Madeleine Simms, *Abortion Law Reformed* (London: Peter Owen Publishers, 1971).

131 Hindell and Simms, *ibid.*, 19.

132 ALRA newsletter, Spring 1970, 8, ALRA Papers, SA/ALR/A11/3, Newsletters, 1963–1971.

133 Hindell and Simms, *op. cit.* (note 130), 117.

134 Hindell and Simms, *op. cit.* (note 130), 177–9.

135 For analysis of the shifting meaning of medical authority in applying the law, see Ellie Lee, Sally Sheldon and Jan Macvarish, ‘The 1967 Abortion Act Fifty Years On: Abortion, Medical Authority and the Law Revisited’, *Social Science & Medicine*, 212 (2018), 26–32.

136

Michelle Jarman , ‘Disability Rights through Reproductive Justice: Eugenic Legacies in the Abortion Wars’, in Russell Shuttleworth and Linda Mona (eds.), The Routledge Handbook of Disability and Sexuality (New York: Routledge, 2020), 132–43.

137 On Germany, see Dagmar Herzog, *Unlearning Eugenics: Sexuality, Reproduction, and Disability in Post-Nazi Europe* (Madison, Wisconsin: University of Wisconsin Press, 2018), 32. On the United States: Greer Donley , ‘Does the Constitution Protect Abortions Based on Fetal Abnormality? Examining the Potential for Disability-Selective Abortion Bans in the Age of Prenatal Whole Genome Sequencing’, Michigan Journal of Gender and the Law, 20, 2 (2013), 291–328 and Ziegler, ‘The Disability Politics of Abortion.’ On Poland: Marta Bucholc , ‘Abortion Law and Human Rights in Poland: The Closing of the Jurisprudential Horizon’, Hague Journal on the Rule of Law, 2022 14, 1 (2022), 73–99 10.1007/s40803-022-00167-9PMC907921840477610.

138 Herzog, *ibid.*, 9.

139 Claire McKinney, ‘Selective Abortion as Moral Failure? Revaluation of the Feminist Case for Reproductive Rights in a Disability Context’, *Disability Studies Quarterly*, 36, 1 (2016), doi https://doi.org/10.18061/dsq.v36i1.3885; Ziegler, ‘The Disability Politics of Abortion’; Shain Neumeier, ‘The Disability Rights Movement Must be Pro-choice’, *Nos Magazine*, 11 April 2017, http://nosmag.org/disability-rights-must-be-pro-choice/; David Perry, ‘Republicans Are Using Fear of Eugenics to Attack Reproductive Rights’, *The Nation*, 4 January 2018, https://www.thenation.com/article/republicans-are-using-fear-of-eugenics-to-attack-reproductive-rights/.

140 Kendall Ciesemier, ‘Leave my Disability out of Your Anti-abortion Propaganda’, *New York Times*, 21 July 2022, https://www.nytimes.com/2022/07/31/opinion/disability-rights-anti-abortion.html.

141 United Nations – Office of the High Commissioner for Human Rights, ‘Stop Regression on Sexual and Reproductive Rights of Women and Girls, UN Experts Urge’, 5 September 2018, https://www.ohchr.org/EN/NewsEvents/Pages/DisplayNews.aspx?NewsID=23503&LangID=E.

142 Herzog, *op. cit.* (note 137), 39.

143 Jarman, *op. cit.* (note 136), 133.

144 Tongue, *op. cit.* (note 19); Garland-Thomson, *op cit.* (note 6); Marsha Saxton , ‘Disability Rights and Selective Abortion’, in Lennard J. Davis (ed.), The Disability Studies Reader, 5^th^ edn. (New York: Routledge, 2017), 73–86 ; Michelle Jarman , ‘Relations of Abortion: Crip Approaches to Reproductive Justice’, Feminist Formations, 27, 1 (2015), 46–66 .

145 For example, Jarman, *op. cit.* (note 136), 48.

146

Alison Piepmeier , ‘The Inadequacy of ‘Choice’: Disability and What’s Wrong with Feminist Framings of Reproduction’, Feminist Studies, 39, 1 (2013), 159–86; 161.

147 Saxton, ‘Disability Rights and Selective Abortion’, 77.

